# Neuronal Innervation of the Subgenual Organ Complex and the Tibial Campaniform Sensilla in the Stick Insect Midleg

**DOI:** 10.3390/insects11010040

**Published:** 2020-01-04

**Authors:** Johannes Strauß

**Affiliations:** AG Integrative Sensory Physiology, Institute for Animal Physiology, Justus-Liebig-Universität Gießen, Heinrich-Buff-Ring 26 (IFZ), 35392 Gießen, Germany; johannes.strauss@physzool.bio.uni-giessen.de; Tel.: +49-641-99-35253

**Keywords:** neurobiology, mechanoreceptors, neuroanatomy *Sipyloidea sipylus*, physiology

## Abstract

Mechanosensory organs in legs play are crucial receptors in the feedback control of walking and in the detection of substrate-borne vibrations. Stick insects serve as a model for the physiological role of chordotonal organs and campaniform sensilla. This study documents, by axonal tracing, the neural innervation of the complex chordotonal organs and groups of campaniform sensilla in the proximal tibia of the midleg in *Sipyloidea sipylus*. In total, 6 nerve branches innervate the different sensory structures, and the innervation pattern associates different sensilla types by their position. Sensilla on the anterior and posterior tibia are innervated from distinct nerve branches. In addition, the variation in innervation is studied for five anatomical branching points. The most common variation is the innervation of the subgenual organ sensilla by two nerve branches rather than a single one. The fusion of commonly separated nerve branches also occurred. However, a common innervation pattern can be demonstrated, which is found in >75% of preparations. The variation did not include crossings of nerves between the anterior and posterior side of the leg. The study corrects the innervation of the posterior subgenual organ reported previously. The sensory neuroanatomy and innervation pattern can guide further physiological studies of mechanoreceptor organs and allow evolutionary comparisons to related insect groups.

## 1. Introduction

Insects have multiple types of mechanoreceptors and other sensilla on body appendages like legs or antenna [1,2,3,4,5,6,7]. The neuronal innervation pattern shows the organization of motor nerves and muscles [8,9] or sensory nerves and their relation to different sensory organs in insect legs [10,11,12,13].

Large internal organs are the chordotonal organs consisting of scolopidial sensilla [14,15], responding to mechanical forces that act on the insect body through substrate vibration, airborne sound, or joint movements, resulting in the stretch or tilting of the dendrites of the bipolar sensory neurons. The femoral chordotonal organ (FCO) and the subgenual organ (SGO) are important chordotonal organs present in all leg pairs [15]. The SGO spans the proximal tibia and is an important receptor for substrate vibrations [16]. In orthopteroid insects, the SGO is accompanied by additional chordotonal organs, forming together the subgenual organ complex [15,17]. At least one further chordotonal organ is usually present just distally of the SGO, termed the distal organ (DO; e. g. in stick insects, cockroaches, locusts) or intermediate organ (e.g., in bushcrickets) [17,18]. These organs respond to substrate vibrations and, in certain insects, also to airborne sound [19]. In stick insects, where the scolopidial sensilla of the SGO (~40) form a hemi-circle in the tibia, the sensilla of the DO (~20) are extending distally from the SGO [18]. A sub-group of the subgenual sensilla is located close to the DO in a cluster, termed the anterior-ventral SGO (avSGO) [18]. By the position of these sensilla which is continuous to the main subgenual organ and the lack of a distinct innervating nerve, these sensilla belong to the SGO [18].

Another important type of mechanoreceptors are the campaniform sensilla (CS) with cuticular caps on the body surface into which the dendrites of sensory neurons insert [14,20,21]. On legs, they detect cuticle strain resulting from leg movements in different directions, muscle contractions, and load increases and decreases, which provide sensory feedback for adaptive movements [22,23,24,25,26,27]. Several groups of CS occur on different leg segments [28,29]. CS also respond to substrate-borne vibrations of low frequencies [30,31], which are transmitted over the leg cuticle [32]. Both chordotonal organs and CS respond to structural deformations by mechanical forces acting on the cuticle or in the body [14].

The mechanoreceptors in stick insects have been studied in detail for their physiology and functional morphology, with a focus on the proximal leg, including the femur [9,33,34,35]. The subgenual organ complex of stick insects was investigated for the neuroanatomy and sensory responses to vibration stimuli [16,18]. The CS in stick insects occur in the proximal tibia in a complex grouping into three separate subgroups (group 6 [29]). These sensilla respond differently to forces on the leg from joint flexion and anterior displacements (proximal CS: Group 6A) or leg extension and posterior displacements (distal CS: Group 6B) [25,27].

The neuronal innervation of tibial CS in relation to the chordotonal organs has thus far not been documented. In other orthopteroid insects, groups of tibial CS are usually closely associated with the larger chordotonal organs, including the SGO [12,30,36,37]. In the stick insect *Carausius morosus* (Sinéty, 1901), the CS are more widely distributed on the proximal tibia in three subgroups [25,29]. Therefore, the innervation of the CS and the chordotonal organs (SGO, DO) is studied here in detail for *Sipyloidea sipylus* (Westwood, 1859) (Phasmatodea: Necrosciinae). This species is a model for sensory neuroanatomy [16,18] due to the light cuticle, which allows documentation of the neuronal structures within the legs. The aim here is to document the nerve innervation of the subgenual organ complex and the tibial CS. The peripheral innervation pattern is analyzed for the spatial relation of the respective nerves, and the influence of distinct sensillum types or the tibial position/proximity of sensilla. In addition, the variation in nerve branches is analyzed since nerve branches of sensory organs occasionally fuse or split in the periphery [17]. These data establish the common neuronal organization of the subgenual organ complex and other mechanoreceptors. The detailed sensory neuroanatomy and innervation patterns can guide further functional studies of mechanoreceptor organs.

## 2. Materials and Methods

### 2.1. Specimens

Adult females of *Siypolidea sipylus* were reared in terraria at the Institute for Animal Physiology, Justus-Liebig-Universität Gießen. The females reproduce parthenogenetically [38]. Animals were kept at 21–23 °C, under a 12:12 light-dark cycle. Individuals were fed with leaves of Rosaceae *ad libitum*. The insects used for neuroanatomical studies were several days after the final molt, and only individuals with intact legs and tarsi were included.

Here, the innervation pattern of the midleg was investigated, which was analyzed in numerous physiological studies [25,26,27,29,39,40,41]. The sensory organization of all leg pairs was very similar [16].

The experiments documented here complied with the principles of animal care of the Justus-Liebig-Universität Gießen, and with the current law of the Federal Republic of Germany.

### 2.2. Leg Morphology

Isolated midlegs were photographed using a Leica 9Si dissection microscope with an integrated camera (1024 × 768 pixels) with the Leica Application Suite version 4.12 (Leica Microsystems CMS GmbH, Wetzlar, Germany).

### 2.3. Scanning Electron Microscopy

For scanning electron microscopy, legs from adult specimens were cut off with scissors at the mid-femur and stored in 70% ethanol (Carl Roth, Karlsruhe, Germany). They were dehydrated in a graded ethanol series (Carl Roth, Karlsruhe, Germany), point-dried (CPD 030, Balzers, Liechtenstein), and mounted with lateral or dorsal sides up on metal holders (Plano, Wetzlar, Germany) [42]. Legs mounted with the dorsal side up were supported using conductive silver cement (Plano Wetzlar, Germany). The legs were sputter-coated with gold (Sputter Coater SCD 004, Balzers, Liechtenstein), and viewed with a LEO 982 SEM (Leo Elektronenmikroskopie GmbH, Oberkochen, Germany). Legs were mounted in different positions, for dorsal view (*n* = 6) and lateral view (*n* = 4). Digital micrographs were stored in tif format (1280 × 1024 pixels).

### 2.4. Staining of Nerves and Sensory Structures by Axonal Tracing

Leg nerves and sensilla were stained by retrograde axonal tracing by infusion with cobalt ions [43]. Prior to staining of the nerves, animals were briefly cold-anesthetized for 5 min at 4 °C. Midlegs were cut off at the coxa-trochanter joint with scissors. Legs were individually fixed in glass dishes covered with Sylgard (Sylgard 184, Suter Kunststoffe AG, Fraunbrunnen, Switzerland) with insect pins, with the ventral side oriented upward, and covered with *Carausius* saline (177.96 mmol NaC1, 17.4 mmol KC1, 25.1 mmol MgC1_2_ × 6 H_2_O, all from Roth, Karlsruhe, Germany; 7.48 mmol CaC1_2_ × 2 H_2_O, from Merck, Darmstadt, Germany; 1.98 mmol Tris, from Sigma-Aldrich, St. Louis, MO, USA; dissolved in *Aqua dest*, pH = 7.4; [44]). The legs' main nerve, nervus cruris [8,44,45], was exposed by cutting and removing the cuticle of the ventral side of the femur with a piece of a blade and removing tendons and muscles with forceps (Dumont #5, Fine Science Tools, Heidelberg, Germany). The nervus cruris was cut close to the femur-tibia joint with iridectomy scissors, and the free end was transferred into a glass capillary filled with a 5% cobalt solution (CoCl_2_ × 6H_2_O, from Merck, Darmstadt, Germany, dissolved in distilled water). Midleg preparations were incubated for 48 h at 4 °C. The intracellular cobalt was precipitated by transferring the legs into a solution of ammonium sulphide (Alpha Aesar, Karlsruhe, Germany; solution of 1% in *Carausius* saline). The legs were rinsed briefly with *Carausius* saline, and fixed in 4% paraform aldehyde (Sigma-Aldrich, St. Louis, MO, USA) for 1 h. They were consecutively rinsed in phosphate buffer (40 mmol Na_2_HPO_4_, 5.74 mmol NaH_2_PO_4_ × 2 H_2_O; both from Merck, Darmstadt, Germany; pH = 7.4), dehydrated in a graded ethanol series (Carl Roth, Karlsruhe, Germany), and cleared in methyl salicylate (Merck, Darmstadt, Germany). Preparations were incubated in methyl salicylate overnight prior to microscopy. Preparations were stored further in methyl salicylate.

Data were obtained for preparations of 44 middle legs from 29 animals. Details in innervation and sensory organ anatomy were analyzed for the anterior innervation (nerve branch T1) for 41 legs and for the posterior innervation (T2) for 44 legs.

### 2.5. Terminology of Nerves and Nerve Branches

The main leg nerve is termed the nervus cruris [8,44,45]. Nerve branches originating from the main nerve were consecutively numbered from proximal to distal in the respective leg segments [9,18,46]. Further nerve branches from the first-order branches were numbered accordingly.

### 2.6. Microscopy and Documentation

Digital photographs were acquired using a Leica DFC 7000 T digital camera (1920 × 1440 pixel; Leica Microsystems CMS GmbH, Wetzlar, Germany) and the Leica Application Suite version 4.9. Most preparations were documented in a series of different focal planes and consecutively stacked with the program CombineZP. The photographs were adjusted for contrast and brightness, and figure panels were assembled and labeled using CorelDraw version 11 (Corel, Ottawa, ON, Canada).

The innervation pattern of the subgenual organ complex and the campaniform sensilla was drawn with a Leitz Dialux microscope (Leitz, Wetzlar, Germany) using a drawing attachment (Leitz), and then digitally redrawn in CorelDraw version 11.

## 3. Results

### 3.1. External Morphology of the Midleg and Location of Campaniform Sensilla

**Figure 1:**
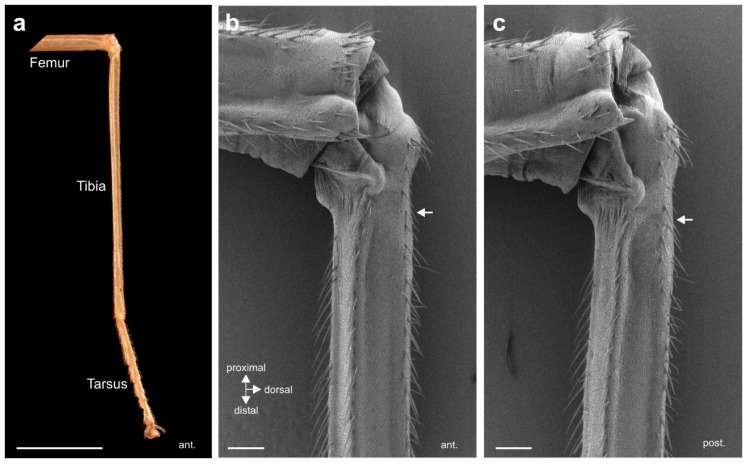
The external cuticle of the *S. sipylus* midleg. (**a**) Photograph of the midleg tibia and tarsus, anterior view. (**b**) Anterior SEM of the cuticle at the femur-tibia joint and proximal tibia. (**c**) Posterior SEM of the cuticle at the femur-tibia joint and proximal tibia. The position of the subgenual organ in the tibia is indicated by white arrows. Scales: (**a**) = 5 mm; (**b**,**c**) = 250 µm.

The morphology of the midleg tibia shows the elongated and slender form characteristic for stick insects (Figure 1a). The external cuticle of the tibia is solid and has almost flat areas with strong cuticular ridges ventrally on both sides, and another ridge medially (Figure 1b,c). There are no apparent structural differences between the anterior and posterior sides.

**Figure 2:**
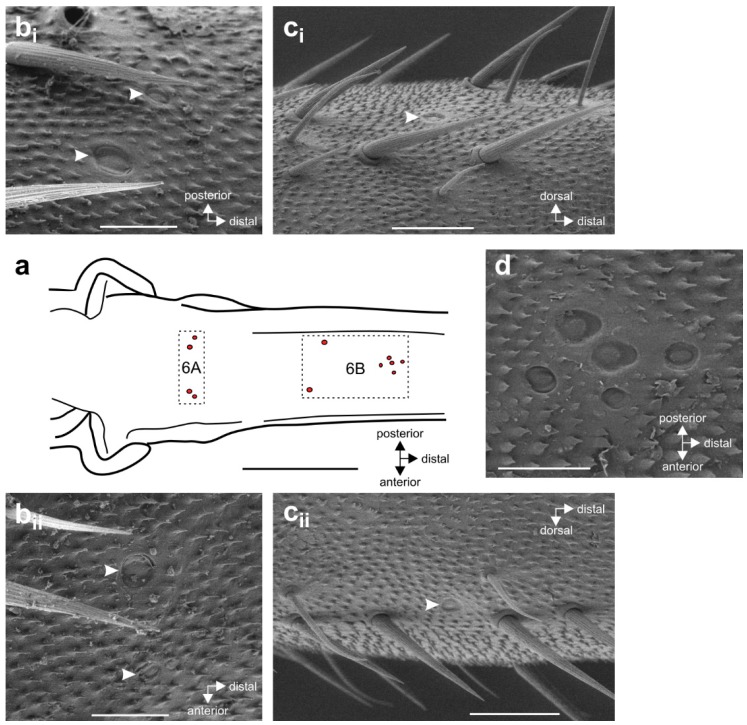
Localization of the tibial campaniform sensilla in *S. sipylus*. (**a**) Schematic of the proximal midleg tibia indicating the position of the tibial CS, groups 6A and 6B, indicated by hatched frames. Cuticular caps are shown in red. (**b**) Group 6A CS in the proximal tibia consists of two posterior (**b_i_**) and two anterior (**b_ii_**) sensilla; cuticular caps of CS are indicated by white arrowheads; dorsal view. (**c**) The single 6B CS on the posterior (**c_i_**) and anterior (**c_ii_**) tibia; lateral view. (**d**) The distal group 6B CS; dorsal view. Scales: (**a**) = 500 µm (**b**,**d**) = 50 µm; (**c**) = 100 µm.

CS have cuticular caps on the leg surface (Figure 2). On the proximal tibia of *S. sipylus*, they form two groups, the more proximal group 6A (Figure 2a,b) and 6B (Figure 2c,d) (the numbering of the CS groups follows reference [29]). Group 6B consists of two sensilla located more proximally (Figure 2a,c) and a group of 5–6 medial sensilla located more distally (Figure 2a,d).

### 3.2. Innervation Pattern of Sensory Elements in the Proximal Tibia

**Figure 3:**
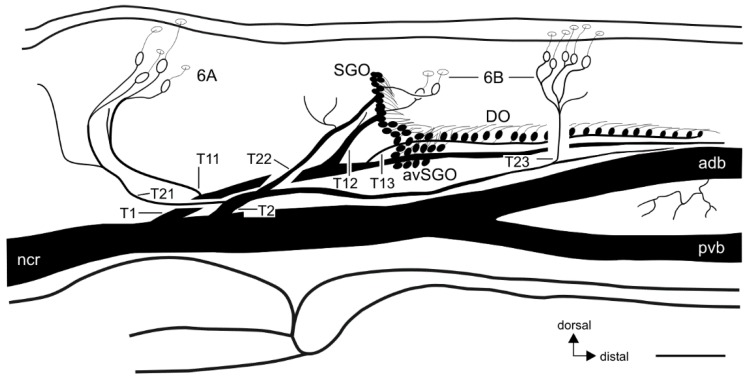
Schematic of the neuronal innervation of the subgenual organ complex and the campaniform sensilla, viewed from the posterior side of the leg. Open nerve branches innervate sensory hairs that are not included in the schematic. Abbreviations: adb, anterior-dorsal branch; avSGO, anterior-ventral subgenual organ; DO, distal organ; ncr, nervus cruris; pvb, posterior-ventral branch; SGO, subgenual organ. Scale = 100 µm.

**Figure 4:**
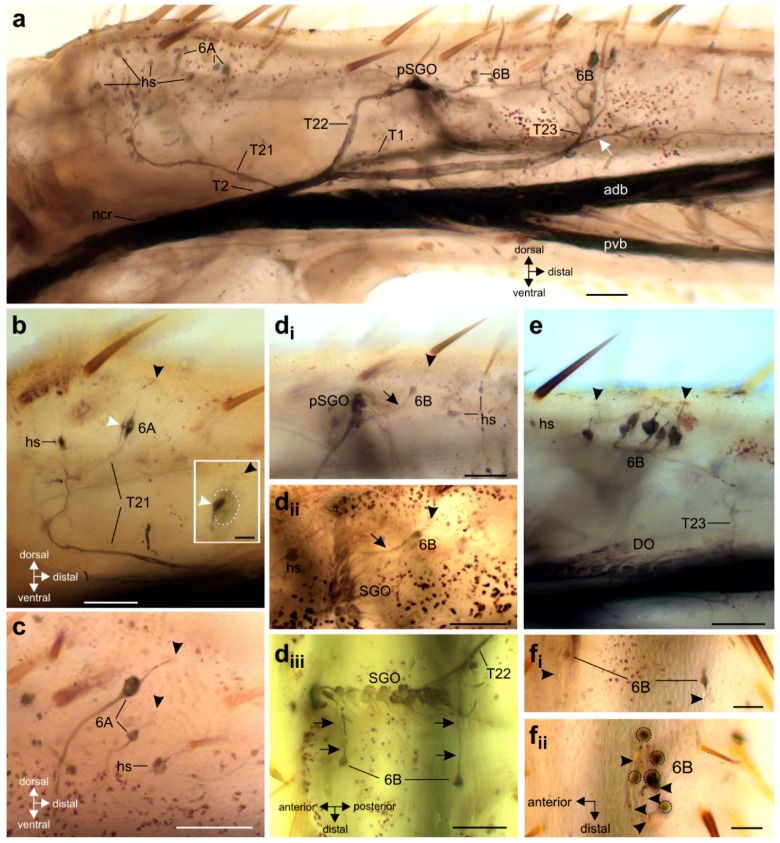
Innervation of the tibial campaniform sensilla. Black arrowheads indicate the cuticular caps of CS. The perspective is a lateral view unless noted otherwise. (**a**) The posterior tibia with sensory elements innervated by nerve branch T2 of nervus cruris (ncr) with successive dorsal branches T21–T23. Note that T2 continues distally of the 6B group of CS (white arrow). (**b**) The posterior 6A CS are innervated by nerve branch T21 (smaller lateral CS indicated by white arrowhead), together with adjacent hair sensilla. Inset shows the soma of the smaller lateral CS, indicated by white arrowhead; the dotted line indicates the outline of the soma of the larger median CS. (**c**) Staining of 6A CS on the anterior tibia. (**d**) The proximal single 6B CS on the posterior (**d_i_**) and anterior (**d_ii_**) tibia is innervated by a nerve branch (arrow) associated with the respective SGO nerve. (**d_iii_**) The dorsal view shows the two separate nerve branches innervating the proximal 6B CS (double arrows) from the two sides of the SGO. (**e**) The larger, distal group of 6B CS at the level of the DO. (**f**) Dorsal view of the 6B CS, (**f_i_**) proximal sensilla, and (**f_ii_**) distal sensilla, with stained somata encircled. The somata are located proximally to the cuticular caps. Abbreviations: adb, anterior dorsal branch; DO, distal organ; hs, hair sensillum; ncr, nervus cruris; pSGO, posterior subgenual organ; pvb, posterior ventral branch; SGO, subgenual organ. Scales: (**a**–**e**) = 100 µm; (**f**) = 50 µm; inset in (**b**) = 25 µm.

The innervation pattern in the proximal tibia was revealed by axonal staining (Figure 3 and Figure 4). The internal chordotonal organs of the subgenual organ complex are located in the region where the CS occur (Figure 3). The chordotonal organs and CS are innervated by two larger nerve branches of the nervus cruris (T1, T2) (Supplementary Figure S1), and each of these branches supplies the sensilla through three successive smaller branches (T11–T13; T21–T23; Figure 3). The most proximal branches (T11 and T21) innervate the two 6A sensilla on their respective sides of the tibia, together with adjacent hair sensilla (Figure 4a–c).

The following branches (T12 and T22) innervate the SGO, with the anterior SGO innervated by T12 and the posterior SGO by T22 (Figure 3 and Figure 4d). Notably, the proximal 6B sensilla are closely associated with the SGO (Figure 3 and Figure 4d). The nerve branches T12 and T22 also innervate the proximal 6B CS located ipsilaterally to the respective nerve (Figure 3 and Figure 4 (d_i_–d_iii_)). The proximal 6B CS are located slightly distally to the SGO sensilla (Figure 3 and Figure 4d).

**Figure 5:**
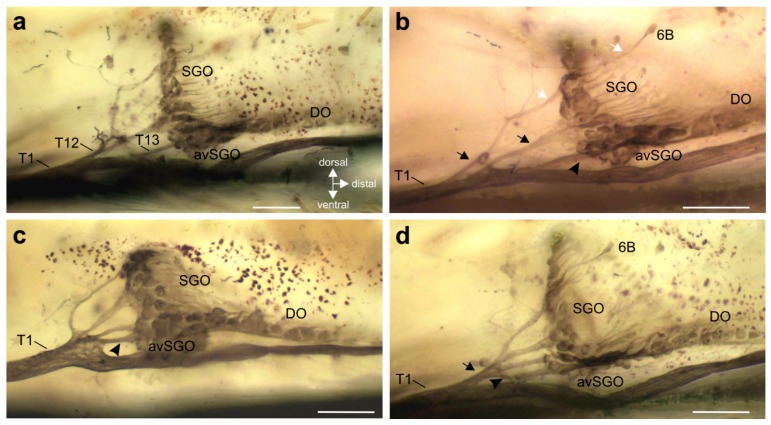
Variation in the innervation pattern of the subgenual organ complex. (**a**) Commonly, the sensilla of the SGO and DO/avSGO are innervated by distinct nerve branches (T12 and T13). (**b**) The SGO can be innervated by two nerve branches (arrows), and an additional branch innervates the DO (arrowhead). White arrows indicate the most proximal, dorsal nerve branch also innervating the proximal 6B CS. (**c**) A joint origin from the tibial nerve branch 1 (T1) for the branches innervating SGO and DO/avSGO (arrowhead) was rarely seen. (**d**) From T1, a common nerve branch for the sensory organs can occur (arrow) from which the branches for SGO, avSGO, and DO (arrowhead) split off. Note that in this preparation, three branches from the common nerve supply the SGO. Abbreviations: avSGO, anterior-ventral subgenual organ; DO, distal organ; SGO, subgenual organ. Scales = 100 µm.

The third nerve branches (T13 and T23) are less symmetrical between the anterior and posterior tibia in their course, and in the sensory elements they supply. T13 is entering T1 closely distally to T12, and supplies the sensilla of the anterior-ventral subgenual organ (avSGO) and the DO (Figure 3 and Figure 5a). The avSGO is not innervated by a separate nerve branch. T23 supplies the distal 6B CS (Figure 3 and Figure 4a,e). The distal 6B group contained, on average, 5 stained CS in tracing preparations (*n* = 23; minimum: 4 CS, maximum: 6 CS). In general, the cuticular cap of a CS is placed slightly distally of the sensillum’s soma (Figure 4b,c,(d_i_,d_ii_),e,(f_i_,f_ii_)). T2 extends further distally in the tibia and innervates sensory hairs (Figure 3). The innervation in fore- and hind-legs is highly similar to the midleg with respect to nerve branches and the sensory organs (not shown).

In sum, the innervation pattern reflects the position of sensory structures rather than the types of sensilla: T1 generally supplies the anterior sensory elements in the proximal tibia, and T2 the posterior ones (Figure 3).

### 3.3. Variation in the Innervation of Sensory Structures

While the overall sensory organization was consistent among the analyzed legs, some variations in the innervation pattern among different legs were notable. This variation usually addressed the occurrence of fused nerve branches, which were commonly separated.

The variation in branching patterns was quantified for five nerve branchings of T1 and T2. In general, the SGO and DO/avSGO were innervated by a single nerve each (Figure 5a; SGO: T12 and DO: T13). The most common variation in the subgenual organ complex was the occurrence of two nerve branches from T1 that both innervated the SGO (Figure 5b; found in 24.4% of preparations; 10 out of 41 preparations). In these cases, the presence of one or two nerve branches to the SGO was always independent of the innervation of the DO, which was innervated by another branch (Figure 5b). The single anterior 6B CS was always innervated by the proximal, most dorsal nerve branch innervating the SGO (Figure 5b). While SGO and DO are usually innervated independently, some variation occurred in the formation of the branches innervating the entire complex: In 82.9% of preparations (34 out of 41), separate nerves for SGO and DO/avSGO leaving T1 were observed (compare Figure 3 and Figure 5a,b). However, in two other preparations (4.9%), the nerve branches innervating the SGO and DO originated together from a swollen part of T1 (Figure 5c). Further, in five preparations (12.2%), a common nerve branch from T1 was found from which the branches innervating the SGO and DO split off (Figure 5d).

For the branches of T1 supplying the 6A and proximal 6B CS, no obvious variation was noted: The nerve branch T11, innervating the proximal hair sensilla, and CS (6A) were always independent of the branches T12 and T13 (*n* = 41).

**Figure 6:**
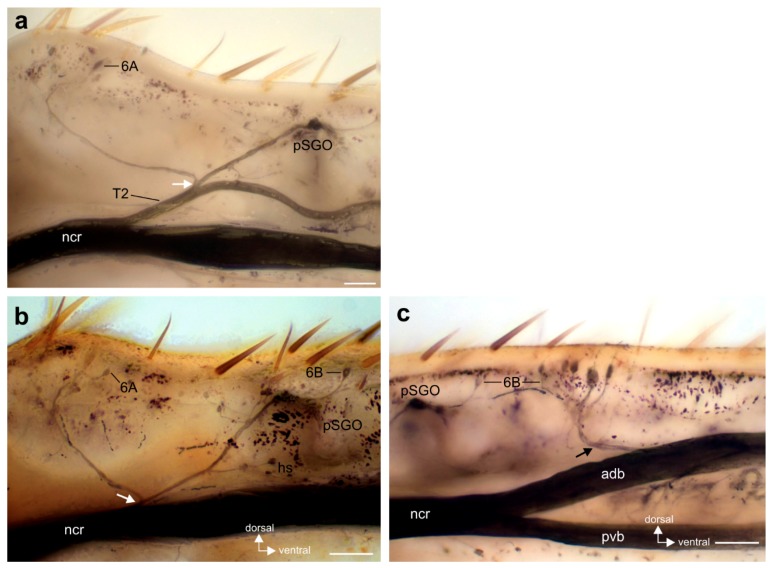
Variation in the innervation pattern of the posterior campaniform sensilla. (**a**,**b**) A common nerve branch for the posterior 6A CS and the posterior SGO can originate from a joint branch from T2 (white arrow). (**c**) The distal 6B CS were innervated from the anterior dorsal branch of nervus cruris (ncr) in one case. The innervation of the 6A and proximal 6B CS in this leg are shown in (**b**). Abbreviations: adb, anterior dorsal branch of nervus cruris; ncr, nervus cruris; pSGO, posterior subgenual organ; pvb, posterior ventral branch of nervus cruris. Scales: (a) = 200 µm; (b,c) = 100 µm.

For the branches of T2, in few preparations, the branches T21 and T22 were not separated, but a single nerve branch split into the two branches supplying the posterior 6A CS as well as the posterior SGO and the posterior 6B CS (Figure 6a,b). The common pattern with separate T21 (posterior 6A) and T22 (proximal 6B, SGO) was found in 39 out of 44 preparations (88.6% of the middle legs). In animals with a common branch innervating 6A and the proximal 6B CS, this was always restricted to only one of the midlegs in cases where both legs were compared (*n* = 4). In one further preparation (out of 44 legs inspected; 2.3%), the nerve branch innervating the posterior SGO and the proximal 6B CS did not extend further distally to innervate the distal 6B CS (Figure 4b), and those were innervated from the anterior-dorsal branch of nervus cruris (Figure 6c). In sum, a common nerve pattern innervating the proximal tibia can be identified (Figure 3), and the variation in nerve branches on this pattern is limited concerning mainly fused nerve branches from the main nerve branches (T1, T2). Notably, the fused nerve branches are rather short (Figure 5d and Figure 6a), and the separate branches to sensory organs are longer after the point of divergence than the fused nerve branch from T1/T2.

## 4. Discussion

### 4.1. Innervation of the Subgenual Organ Complex and the Campaniform Sensilla in the Proximal Tibia

In stick insects, important mechanosensory organs occur in all segments of the thoracic leg pairs [9,16,28,29]. The present study analyses the neuronal innervation of the subgenual organ complex and 6A/6B CS in the proximal tibia. Axonal tracing shows that the subgenual organ complex and the 6B CS are placed at a similar level of the tibia. The 6A CS are placed more proximally and innervated together with proximal hair sensilla. The CS (6A and 6B) in the tibia have been investigated in *Carausius morosus* (Lonchodinae) for their functional morphology and physiology [25,29]. The distribution of CS in *S. sipylus* is highly similar with respect to position and number of sensilla. In the distal 6B CS, the caps have round shapes and are placed towards the proximal margin of the cuticular collar, a morphology also described in *C. morosus*, and discussed as a morphological basis for the observed directional responses to loads applied in different directions [27]. Notably, in the distal 6B group, the most proximal and distal caps are located medially in a cross-shape, while there is no central cap in *C. morosus* [29].

Overall, the documented innervation of sensilla in *S. sipylus* depends on the position rather than the sensillum types, as sensilla in close proximity are innervated together. This was found for CS and hair sensilla (T11, T21, and T23) and CS and scolopidial sensilla (T12, T22). The innervation pattern reflects both the proximo-distal and the anteroposterior position of sensilla. The distal 6B CS with size differences of the caps [27] are jointly supplied by T23. In *S. sipylus*, the influence of the position on the innervation pattern is prominent along the proximo-distal axis due to the separate groups of CS, in comparison with related insect groups: In cockroaches, the tibial CS are in closer proximity with each other [23,30] and are innervated by one nerve branch together with the SGO [30], while in the locust, less CS occur in the proximal tibia [12].

In *S. sipylus*, the posterior subgenual organ is innervated by T22 and contains sensilla, which are continuously organized with the adjacent sensilla of the subgenual organ innervated by T12. It is, therefore, not possible to count the sensilla separately that are innervated by the distinct nerve branches T12 and T22 [18]. At the posterior side of the SGO, there is no indication of the presence of the accessory organ. This small chordotonal organ is usually located close to the posterior subgenual organ and to one or a few posterior CS in several groups of orthopteroid insects, but it is missing in others such as locusts [47,48]. The scolopidial sensilla of the accessory organ can usually be distinguished by small sensory neurons and a proximal orientation of the dendrites [47].

Overall, the CS in the proximal tibia in *S. sipylus* is closely associated with placement and in innervation with chordotonal organs. From a comparative perspective, this is similar to Orthoptera [12,36,37,49], where, however, the largest group of tibial CS is located close to the subgenual organ [12,37,50]. This is notably different in stick insects, where the largest group 6B is placed ~400 µm more distally at the level of the DO, and innervated independently from the chordotonal organs.

### 4.2. Variation in the Neuronal Innervation

While some variation in the sensory innervation is notable, an innervation pattern that is most common can be identified (Figure 3). The observed variation in innervation concerns the proximo-distal order of nerve branches (Figure 5 and Figure 6). The common innervation pattern, however, was found to be robust (present in >75% of preparations for different branching points). The nerve branches T11 and T21 from the 6A CS and sensory hairs had the lowest variation ratios. A variation occurred more often for the sensilla which are in close proximity, especially from the SGO, either by forming a second SGO branch or by fusion with the DO branch. This implies that the innervation reported for *S. sipylus* and *C. morosus* with a single branch from T2 for the posterior hair sensilla and the posterior SGO [18] constitutes an exception from the more common pattern with the two nerves separated (T21, T22).

Obviously, the innervation pattern is not strictly determined, and sensory nerves are organized during embryonic development from the peripheral sensilla, which sends axons towards the central nervous system [51,52]. This growth direction depends on morphogens in the limb bud [53,54]. It is likely that the observed variation in the peripheral nerves—like the fusion of nerve branches from T1 or T2—has no functional correlation, as this depends on the axonal projection into specific neuropils in the central nervous system (discussed below). Notably, no variation exchanging the innervation between the anterior and posterior sides of the tibia was observed, which indicates a mechanism to separate axonal directions in this leg axis during embryogenesis. Hence, common or separate positions in the leg may also sort the axons in the periphery and set up the central sensory projections.

### 4.3. Functional Aspects of Innervations Considering the Central Projections

The spatial organization of sensilla and the innervation pattern in the periphery can influence the central projection of sensory afferents. Sensory neurons project into specific neuropils of the central nervous system [55,56], though the determination of projections can depend on the peripheral position (somatotopy) or the sensillum types (modality specificity). Somatotopic projections were shown for leg sensory hairs [57,58,59] and chordotonal organs [13,60,61]. For mechanoreceptors (CS and hair plates) in locusts, sensilla from similar locations share similar central projections that are not largely influenced by the specific sensillum types [62]. However, in the proximal leg, projection areas correlate with the different sensillum types [11].

The tibial CS respond differentially to directed force increases/decreases muscle forces [25,27] but are not involved in sensory feedback by the CS on more proximal leg segments that control the coxal protractor and retractor muscles [29]. Notably, the 6B CS and CS from other leg segments in *C. morosus* were shown to form overlapping projections in the central nervous system [29]. Likely, the 6A CS also share the similarities in central projection. Those are also similar to projections of other leg mechanoreceptors like hair sensilla in *C. morosus* [63]. That similarity has been interpreted as an input system to pre-motor interneurons over distinctly separated sensory units [29,33]. The SGO/DO projections have thus far not been documented for stick insects. The central projections of afferents from 6A CS as well as the vibrosensitive SGO [16] will complement the data on sensory inputs and their specificity. Another topic concerning the neuroanatomy is the developmental organization of the innervation pattern, especially its molecular mechanisms, which has thus far not been analyzed in stick insects.

## 5. Conclusions

This study shows the close association of nerve branches innervating the tibial CS and the subgenual organ complex, as well as some variation in the innervation. Overall, the shared innervation of sensilla depends on their position rather than on sensillum types, as is highlighted by the innervation of the anterior and posterior SGO together with the single proximal CS located ipsilaterally. The distal 6B sensilla, located more distally from the SGO, are innervated distinctly from the chordotonal organs. The innervation data comparing several preparations show the variation in nerve branches and correct the previously reported innervation of the posterior tibia. The sensory neuroanatomy and innervation pattern can guide further functional studies of mechanoreceptor organs, e.g., for the central projections of different afferents and the consecutive processing of sensory inputs. They also allow comparisons with other insect groups to infer adaptive changes in the mechanoreceptor system.

## Supplementary Materials

The following are available online at https://www.mdpi.com/2075-4450/11/1/40/s1, Figure S1: Innervation of the anterior and posterior tibia from nervus cruris.
