# Supplementary material for: Neuronal Innervation of the Subgenual Organ Complex and the Tibial Campaniform Sensilla in the Stick Insect Midleg
Source: Insects. 2020 Jan 4;11(1):40. doi: 10.3390/insects11010040 (PMC7022571; doi:10.3390/insects11010040)
Supplement: Supplementary file 1 [file insects-11-00040-s001.pdf]

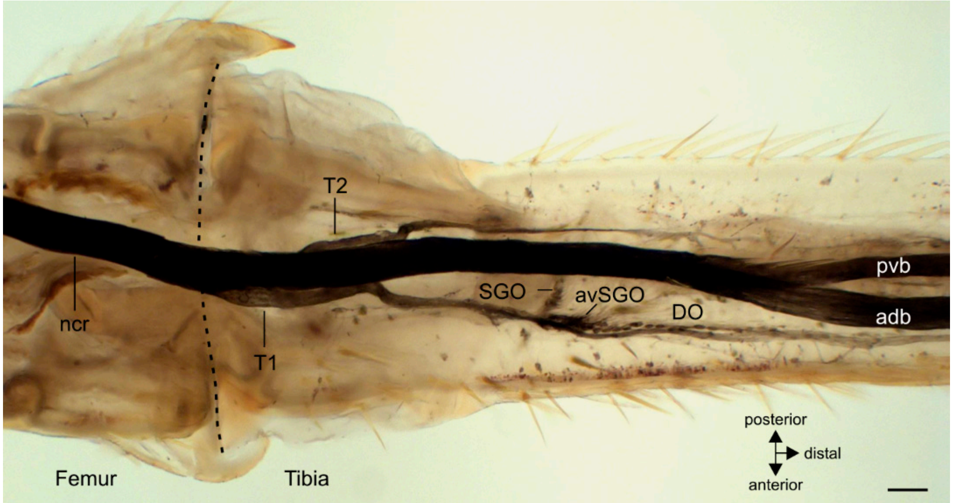

Supplementary Figure 1. Innervation of the anterior and posterior tibia from nervus cruris. Ventral view of the nerve branches from nervus cruris in the proximal tibia: T1 at the anterior side, T2 at the posterior side. Hatched line indicates the border between femur and tibia. Scale bar = 100 µm.

Abbreviations: adb, anterior dorsal branch; avSGO, anterior-ventral subgenual organ; DO, distal organ; ncr, nervus cruris; pvb, posterior ventral branch; SGO, subgenual organ.
